# Retraction Note: Integrated analysis of gene networks and cellular functions identifies novel heart failure biomarkers

**DOI:** 10.1186/s41065-026-00751-1

**Published:** 2026-10-16

**Authors:** Jiang Juncheng, Chen Lei, Lin Hao, Liang Fei

**Affiliations:** Department of Cardiovascular Surgery, Shandong Provincial Hospital, Shandong Fist Medical University, No. 324, Jingwu Road, Jinan, 250021 China

**Retraction Note: Hereditas 162, 152 (2025)**

**https://doi.org/10.1186/s41065-025-00521-5**

The Editor-in-Chief has retracted this article. After publication, concerns were raised regarding the in vitro images presented in Figs. 7 and 8 of this article, where the patterns of cells and colonies do not appear consistent with natural cell growth and proliferation. Figure 7D OE-COL9A1-AC16 and OE-MTIF3-AC16 images also appear highly similar. Additionally, the uncut western blot gel presented in the Supplementary Material appears to show contradictory results, with four proteins probed in adjacent lanes with different results. It has also been noted that a number of references in this article do not appear to support the statements preceding the citations, and the cell line descriptions in the Methodology section are unclear or misleading.

The Editor-in-Chief therefore no longer has confidence in the presented data.

The authors have not responded to correspondence regarding this retraction.

